# Therapeutic Options for Recurrence of Primary Focal Segmental Glomerulonephritis (FSGS) in the Renal Allograft: Single-Center Experience

**DOI:** 10.3390/jcm10030373

**Published:** 2021-01-20

**Authors:** Kalliopi Vallianou, Smaragdi Marinaki, Chrysanthi Skalioti, Sophia Lionaki, Maria Darema, Christina Melexopoulou, Ioannis Boletis

**Affiliations:** Nephrology and Kidney Transplantation Clinic, Laiko General Hospital, National and Kapodistrian Universtity of Athens, 11527 Athens, Greece; smaragdimarinaki@yahoo.com (S.M.); c_skalioti@yahoo.com (C.S.); sofia.lionaki@gmail.com (S.L.); mdarema0@gmail.com (M.D.); xmelexopoulou@gmail.com (C.M.); laikneph@laiko.gr (I.B.)

**Keywords:** kidney transplantation, FSGS recurrence, outcomes, plasmapheresis, therapeutic agents

## Abstract

Focal Segmental Glomerulosclerosis (FSGS) recurrence after kidney transplantation (KTx) is relatively frequent and is associated with poor graft survival. The aim of this study was to investigate which management strategies were associated with better outcomes in our cohort of KTx recipients with primary FSGS. We retrospectively collected data on patients with primary FSGS who received a KTx between 1993 and 2019. A history of biopsy proven FSGS in native kidneys and new onset of significant proteinuria early post-KTx led to the diagnosis of recurrence, which was confirmed by graft biopsy. From 1993 to 2019 we performed 46 KTxs in patients with primary FSGS. We identified 26 episodes of recurrence in 25 patients, 67% of them occurring in males. They were younger at the time of KTx (33.8 vs. 41.1 years old, *p* = 0.067) and had progressed to end stage renal disease (ESRD) faster after FSGS diagnosis (61.4 vs. 111.2 months, *p* = 0.038), while they were less likely to have received prophylactic plasmapheresis (61.5% vs. 90%, *p* = 0.029). 76.7% of recurrences were found early, after a median of 0.5 months (IQR 0.1–1) with a median proteinuria was 8.5 (IQR 4.9–11.9) g/day. All patients with recurrence were treated with plasmapheresis, while 8 (30.7%) additionally received rituximab, 1 (3.8%) abatacept, and 4 (15.4%) ACTH. 7 (27%) patients experienced complete and 11 (42.3%) partial remission after a mean time of 3 (±1.79) and 4.4 (±2.25) months, respectively. Prognosis was worse for patients who experienced a recurrence. Eleven (42.3%) patients lost their graft from FSGS in a median time of 33 (IQR 17.5–43.3) months. In this series of patients, primary FSGS recurred frequently after KTx. Prophylacic plasmapheresis was shown efficacious in avoiding FSGS recurrence, while timely diagnosis and plasmapheresis-based regimens induced remission in more than half of the patients.

## 1. Introduction

Glomerulonephritis is the primary cause of end stage renal disease (ESRD) in 10.5–34.5% of patients [1] and relapse rates after kidney transplantation (KTx) are as high as 2.9–12% in various registries [2]. Focal segmental glomerulosclerosis (FSGS) accounts for 2–4.6% of the primary diseases found in adults and 11–12.9% found in children receiving a kidneyallograft [2,3]. Focal segmental glomerulosclerosis recurrence in the graft remains a major challenge, as it occurs early with the reported incidence accounting for 30–50% in adults [3,4,5,6,7] and 60% in children [6,7,8]. It often responds poorly to treatment, with a substantial proportion, 30–50% of these patients losing their graft within 5 to 7 years [3,5,7,9,10,11,12].

Risk of recurrence has been associated with younger age at diagnosis of FSGS and KTx, an aggressive disease course in the native kidneys and previous graft loss due to FSGS [9,11,13,14,15]. The latter is the strongest predictor of recurrence, with a risk of 65–85% in the second transplant [9,10,11]. Others report factors such as non-black race, graft from a living donor, and native kidney nephrectomy, but evidence is controversial [4,6,7]. Duration of renal replacement therapy and choice of immunosuppression do not seem to affect risk of recurrence [4,6,12].

In the pathogenesis have been implicated one or more circulating permeability factors which are thought to cause podocyte injury, including soluble urokinase plasminogen activator receptor (supar) and/or antibodies binding the CD40 molecule [13,14,15,16]. As a result, treatment of recurrence focuses in removing this factor from the circulation, and thus most centers use therapeutic plasmapheresis or immunoadsorption, alone or in combination with rituximab, with satisfactory results [4,17,18,19], while perioperative preventive plasmapheresis [19,20,21,22] has also been used. Other treatment options are not routinely used due to conflicting findings or serious adverse effects [17,22,23,24,25,26,27,28].

We aimed to investigate which management strategies of FSGS recurrence in the graft were associated with better outcomes in our cohort of KTx recipients with FSGS as primary disease.

## 2. Materials and Methods

We retrospectively collected data of patients with a history of biopsy proven FSGS in their native kidneys transplanted between 1993 and 2019. Our study complied with the Declaration of Helsinki and the appropriate approval of our Data Protection Officer was warranted (No. 81/30-10-2020).

Patients with FSGS were initially identified; those with familial or secondary FSGS were excluded. Familial FSGS was diagnosed by family history and identification of the gene mutations responsible, and secondary FSGS by medical history (e.g., hypertension, obesity) in association with native kidney biopsy findings and natural course of the disease. Occasional patients with unknown native kidney disease received the diagnosis of primary FSGS due to early recurrence in the graft. All patients had a minimum follow up time of one year after KTx.

Recurrence was defined as new onset of persistent proteinuria above 3 g per day measured by 24-h urine collection. Other causes of proteinuria were excluded by allograft biopsy. Light microscopy revealed either FSGS lesions or normal glomeruli, especially if performed early after recurrence, whereas electron microscopy showed podocyte foot process effacement. Recurrences, which developed within the first three months after KTx were characterized as “early”, while “late” were characterized if they occurred afterwards. Complete remission was defined as reduction of proteinuria below 0.5 g per day and partial remission as reduction by 50% or more of the initial value.

Induction immunosuppression for KTx included therapy with antithymocyte globulin or an anti CD25 monoclonal antibody (daclizumab or basiliximab). The immunosuppressive regimen consisted of a calcineurin inhibitor (tacrolimus or cyclosporine A), mycophenolate mofetil or mycophenolate sodium and steroids. Cyclosporine A 2-h target levels were 700–900 mg/dL for the first-year post-transplant and 500–600 mg/dL afterwards, while target tacrolimus trough levels were 8–10 ng/dL and 6–8 ng/dL, respectively. A pulse of 500 mg intravenous methylprednisolone was administered during surgery, followed by 20–40 mg daily for the first two weeks and gradual tapering to the maintenance dose of 4 mg/day after three months. Delayed graft function was defined as the need for dialysis during the first week post-ΚΤx.

Prophylactic plasmapheresis protocol consisted of three sessions prior to KTx and three sessions afterwards for recipients from a living donor, while patients who transplanted from deceased donors received three sessions every other day post-operatively. Documentation of FSGS recurrence was followed by a new therapeutic scheme of plasmapheresis, which began as soon as possible and consisted of 1.5 times plasma volume exchange, replaced by a human albumin solution 5%. In this scheme, three daily sessions were followed by a three-times-a-week schedule for three consecutive weeks and then treatment was individualized by each patient response and outcome.

Rituximab was administered in two doses of 1 g each, two weeks apart and abatacept in two doses of 10 mg/kg each, two weeks apart as well. When combined with plasmapheresis, rituximab was always administered after a session and 24 to 48 h before the next one, in order to minimize drug removal during the procedure; no additional dose was necessary. The usual dose for ACTH was 100 IU twice a week and minimum duration of treatment was four months. We administered the synthetic analogue tetracosactide intramuscularly.

Long standing clinical experience in the field led us to the usage of the aforementioned protocol, while we generally tend to prefer induction with antithymocyte globulin in this group of patients. Urine protein excretion was measured daily for the first two weeks, weekly for the next month, every two weeks for one more month, monthly until one year was completed and thereafter every three months. When recurrence occurred, we performed a graft biopsy and began therapeutic plasmapheresis as soon as possible. We also administered rituximab early after diagnosis. In patients who did not respond or become plasmapheresis dependent we subsequently added treatment with ACTH, as described above.

### Statistical Analysis

Categorical variables are expressed as frequencies (percentage) and numerical data as mean (±SD) or median (IQR). Differences were calculated using chi-square or Fisher’s exact test for categorical data, Student’s *t*-test and ANOVA for normally distributed numerical variables and Mann–Whitney-U and Kruskal–Wallis test for skewed numerical variables. Graft survival curves were performed with the Kaplan–Meier method and differences with log-rank test. Significance was defined as *p* < 0.05. Statistical analysis was performed using Microsoft Excel (Microsoft Office 2007) and STATA software (STATA 13 for Windows, StataCorp, College Station, TX, USA).

## 3. Results

From 1993 to 2019, 66 patients with FSGS were identified. 20 of them with familial or secondary FSGS were excluded. 43 patients with primary FSGS, receiving 44 kidney allografts in our hospital, were included in our analysis. Two more patients, with unknown primary renal disease, were diagnosed with FSGS early post-transplant, with severe proteinuria and typical findings in allograft biopsy. In total, out of 46 kidney transplants, 26 FSGS recurrences were documented (57%).

### 3.1. Patient Characteristics

Table 1 displays the characteristics of the FSGS cohort, including those with and without recurrence; 65% of patients in both groups were males. In those who experienced recurrence of FSGS, the initial disease diagnosis in their native kidneys occurred earlier in life (21.8 vs. 27.7 years), run a more aggressive disease course and led to ESRD and KTx at a younger age (33.8 vs. 41.1 years). Indeed, time from FSGS diagnosis to ESRD in the recurrence group was 61.4 months, significantly shorter than in the non-recurrence group (111.2 months, *p* = 0.038). They also received a graft from a deceased donor more often, while delayed graft function was more frequent. However, donor age and time on renal replacement therapy were similar in both groups. Two patients in the recurrence group had received a second graft and one more a third one; notably, among these patients all previous graft losses were attributed to FSGS recurrence. Only one of these previous transplants are included in our study, since the rest did not take place in our transplant center. On the contrary, all patients without recurrence were first kidney transplant recipients.

### 3.2. Treatment Options and Efficacy

**Figure 1:**
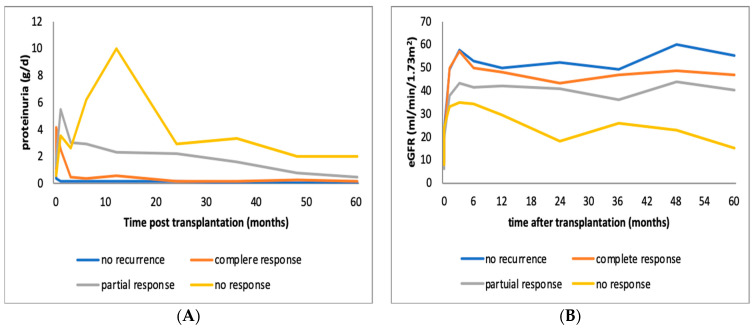
(**A**) Alterations of mean proteinuria in patients with FSGS recurrence in the graft whoexperienced no response, complete or partial remission following immunosuppressive therapy. (**B**) Alterations of mean estimated GFR in patients with FSGS recurrence in the graft who experienced no response, complete or partial remission following immunosuppressive therapy. eGFR: estimated glomerular filtration rate, FSGS: focal segmental glomerulonephritis.

Patients with recurrence were divided into three subgroups, those who showed complete remission following therapy, those with partial remission and those without response (Table 2). A total of 7 out of 26 (27%) patients achieved complete remission in an average of 3 (±1.7) months, 11 (42%) patients achieved partial remission in 4.4 (±2.25) months, and 8 (31%) did not respond to treatment. The majority of patients (75–80%) experienced early recurrence, most of them within days after KTx and few (20–25%) later on, after the first three months. However, one patient in the complete remission and three in the partial remission group suffered both an early and a late recurrence, the early within the first month post-KTx and the late one 12 to 14 months after remission and treatment discontinuation. Higher proteinuria levels were associated with worse response to treatment (6.1 vs. 9.5 vs. 10.3 g/day). Figure 1 depicts proteinuria and creatinine level changes in the course of the disease.

Prophylactic plasmapheresis was shown to be the most important intervention for avoiding FSGS recurrence in the graft in this cohort. Prophylactic plasmapheresis showed a favorable effect on risk of recurrence, as 90% of non-recurrent patients had received the prophylaxis protocol compared with 62% of recurrent patients (*p* = 0.029). However, prophylactic plasmapheresis was not found to correlate with milder recurrence and better response to treatment post-recurrence.

Regarding therapeutic plasmapheresis, a median of 16 (IQR 14–19) sessions were applied until achievement of sustained remission in the complete remission group, while more sessions were needed for the partial remission group (median 49, IQR 24–61); in fact, four patients became plasmapheresis dependent and required additional treatment to achieve plasmapheresis weaning. Two of these patients underwent more than 200 sessions in total, until they could be weaned-off. In the no remission group, after a median of 14 sessions (IQR 12–31), we discontinued plasmapheresis, accepting either non-responsiveness to treatment or progression to non-reversible chronic kidney disease.

Rituximab, as additional therapy, was administered in 8 out of 26 patients with recurrence; 7 of them achieving complete or partial remission. Four patients with refractory disease received ACTH; one showed complete remission, two patients achieved partial remission, while a third one had no response. However, ACTH administration was associated with significant adverse effects: one patient developed diabetes mellitus in need of insulin treatment, one crusted scabies and the remaining two had regular episodes of mild hypokalemia. Abatacept was given in one patient with severe nephrotic syndrome, resistant to any therapy, and failed as well.

### 3.3. Outcomes

**Figure 2:**
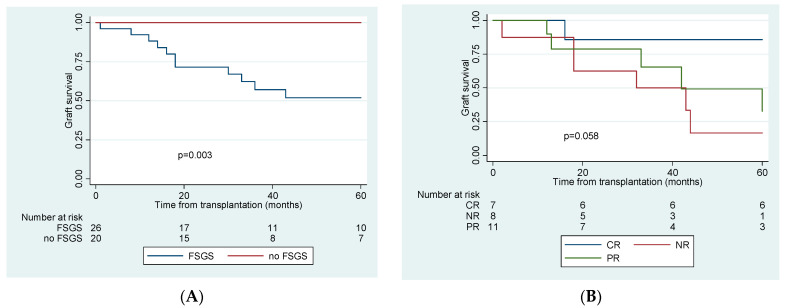
Kaplan–Meier 5-year graft survival curve comparing (**A**). patients with and without recurrence of FSGS post-transplantation and (**B**). patients with complete, partial and no response to treatment of recurrence. CR: complete remission, PR: partial remission, NR: no remission, FSGS: focal segmental glomerulonephritis.

FSGS recurrence was associated with worse graft and patient outcomes (Table 1 and Table 2). Recipients without recurrence had better renal function one year post-KTx (eGFR 53 vs. 45 mL/min). During a median follow-up of 33.5 months (IQR 23–57.8), no graft was lost among patients without recurrence, compared to 15 graft losses (58%) in the recurrence group, during a median follow-up of 33 months (IQR 18.2–140.6). A total of 11 grafts failures (73.3%) were attributed to FSGS recurrence, one patient died of acute myocardial infarction with a functioning graft, one had an acute rejection episode, and two more patients lost their graft due to chronic rejection. Fourout of elevenpatients (36.4%) with partial response to treatment and 7 out of 8 patients with no response experienced graft loss in a median time of 33.1 (IQR 17.9–57.7) and 18.3 (IQR 15.4–73.2) months, respectively. The eighth patient with non-responsive FSGS has still a functioning graft with an eGFR of 17 mL/min at 32 months post-KTx. Overall, five-year graft survival in different groups is depicted in Figure 2.

In addition, adverse effects regarding ACTH use, recurrence treatment was considered generally safe. Patients on rituximab and plasmpapheresis all received Pneumocystis prophylaxis with cotrimoxazole or alternate agents; no case of Pneumocystis pneumonia was reported. Treatment was implicated by threecases of microbial infections associated with central venous catheters, followed by appropriate antimicrobial treatment and catheter removal that did not substantially delay recurrence treatment.

## 4. Discussion

In this cohort of KTx recipients with ESRD due to primary FSGS, a recurrence rate of 56% was recorded. One third (27%) of them, achieved complete remission after appropriate treatment, 42% partial remission and the remaining 31% showed no response. Five-year graft survival after recurrence was 52%, dropping to 17% in non-responders. These results are similar or slightly worse to those previously reported [5,12,17,29]. However, better outcomes have been observed in cohorts that included patients with genetic or secondary forms of FSGS who rarely experience disease recurrence [5,17,30]. Moreover, definitions of recurrence, complete and partial remission differ among studies, making comparisons of results difficult.

The most consistent predictors of FSGS recurrence have been younger age at diagnosis and a shorter course from primary disease diagnosis to ESRD [4,6,7,9,31]. We also found that patients with recurrence were younger both at the time of diagnosis of FSGS in native kidneys (21.8 vs. 27.7) and at KTx (33.8 vs. 41.1) and had a more rapid progression to ESRD after diagnosis (61.4 vs. 111.2 months). Only the last factor, though, was of statistical significance between the two groups in our case. Another strong predictor of FSGS recurrence in the graft is loss of previous grafts due to FSGS [3,5,6,9]. Notably, one of our patients, who received a second and later a third graft, experienced a recurrence in both KTxs and lost them despite intensive treatment. Another patient, transplanted for the second time developed an early recurrence, responded well to treatment, and maintained remission for one year. Unfortunately, both patients lost their first graft due to FSGS recurrence. On the contrary, we recorded no previous transplants in the non-recurrence group.

Additional risk factors that have been described by others include living donor source, native kidney nephrectomy, and non-black race [4,6,7,32,33]. The initial observation that living donation is associated with higher recurrence rates [13,33] was not supported by further evidence and could be attributed to the fact that younger patients, who were more likely to have a living donor, tend to recur more often [9,12,34]. However, living-donor grafts may lose their survival advantage over deceased-donor grafts, as a consequence of FSGS recurrence and treatment failure [6]. Donor source was not a risk factor for recurrence in our cohort. Of note, living donors were numerically more frequent in the recurrence group. Non-black race has also been associated with worse outcomes in several studies; genetic factors could explain this observation [4,7,24]. In Greece, our experience with non-white patients is limited; in our cohort there was only one African-American recipient, who has shown no signs of recurrence.

Interestingly, we found that delayed graft function (DGF) was more common among patients with FSGS recurrence (38.5 vs. 15%). Higher rates of deceased donor donation in this group partially explain this association (9 out of 13 patients). On top of that, DGF is thought to be a consequence of immediate, severe proteinuria, when FSGS recurs as early as hours or few days post-KTx [6,34] and has been described as a major independent predictor of graft loss in these patients, regardless of graft source. Interestingly, graft failure from recurrent FSGS was seventimes more common among pediatric KTx recipients with DGF [6].

Importantly, prophylactic plasmapheresis showed a favorable effect on risk of recurrence, although it was not found to correlate with milder recurrence and better response to treatment post-recurrence. The first study reporting the use of prophylactic plasmapheresis was in children with primary FSGS and showed encouraging results, with 33% recurrence rate in patients who received prophylaxis, compared to 67% in those who did not [24]. Further research showed a lower recurrence rate [20] or a more benign course and better response to treatment [20,35]. However there were studies with disappointing results regarding prophylactic plasmapheresis [22,36,37]; nonetheless in one of them, though plasmapheresis failed to prevent recurrence, rituximab showed a moderate protective, yet not statistically significant effect [36]. In our practice, we have applied a prophylactic protocol of plasmapheresis in these patients since 2004 and we have gathered substantial clinical experience, as already published by Lionaki et al. in 2015 [38].

Beyond its use in prevention, plasmapheresis has also evolved as the cornerstone treatment for FSGS recurrences in the graft since its first implementation in these patients in 1985 [39]. The rationale is to remove the toxic circulating factors. Several techniques have been used, including immunoadsorption with protein A, which has been proven equally effective and probably with better safety profile [38,40]. It is now established knowledge from studies performed in large cohorts that 70% of patients, either adults or children [18], achieved complete or partial remission. Heavy proteinuria and delay in treatment initiation after recurrence onset have been associated with worse response to treatment [17,18]. Likewise, all of our patients who experienced a recurrence received therapeutic plasmapheresis immediately after diagnosis and the response rate was as high as 73%.

Furthermore, since 2000, rituximab has been widely used in FSGS recurrence as adjuvant to plasmapheresis. Rituximab depletes B lymphocytes and also enhances regulatory T cell activity; T cell malfunction is believed to play a major role in pathogenesis of proteinuria [17,41,42]. Moreover, there is evidence of non-immunological actions since rituximab directly modulates podocyte function by preventing the disruption and remodeling of actin cytoskeleton [42]. An overall response rate of 58 to 79% with the use of rituximab has been reported in such patients [22,41,42,43]. Although different treatment modalities have been used and it is not always clear which one contributed most to remission, it is probable that the combination of plasmapheresis and rituximab is currently the most promising regimen [3,4,20].

Routine use of combined immunosuppressants do not seem to affect recurrence risk [4,5,35]. Cyclosporine A, in very high intravenous or oral doses (two-hour peak levels of 1.200–1.400 mg/dL), has been part of treatment protocols that showed satisfactory results [27,29]. The potential nephrotoxicity of cyclosporine however remains a major concern precluding its use in our practice.

Abatacept is a fusion protein that acts as a co-stimulatory inhibitor of B7.1. B7.1 has been detected on podocytes of proteinuric patients and is considered essential for T cell activation [43]. Interest was drawn on abatacept in 2013, when Yu et al. successfully treated five patients with resistant FSGS, four of them with recurrent and one with primary disease. All of them had positive B7.1 staining in biopsy specimens [44]. However, a case-series of nine patients [28] and a number of case reports [45,46] published later, showed no effect on refractory recurrent FSGS after KTx. We administered abatacept in one patient with severe refractory disease who showed no response. A recent review suggests administration of abatacept only in patients with positive B7.1 biopsy staining [47]; in our patient’s case no such staining was available.

Adrenocorticotropic hormone (ACTH) is an old agent that has been repeatedly used in the treatment of steroid-resistant nephrotic syndrome [48]. ACTH stimulates cortisol excretion and, more importantly, activates melanocortin receptors located on podocytes, directly reducing oxidative stress, and possibly regulating cytoskeleton function [49,50,51]. ACTH has recently resurfaced as a promising rescue therapy in nephrotic syndrome. A review demonstrated response rates of about 40% in refractory FSGS and membranous nephropathy [48]. Two recent case-series report comparable remission rates (between 34 and 50%) when ACTH was used to treat FSGS recurrence in KTx recipients non-responsive to other treatments [25,26]. Four of our patients received ACTH, one experienced a spectacular complete remission within three weeks, two plasmapheresis dependent patients responded partially, allowing plasmapheresis weaning and one showed no response. Although adverse events were frequent, as mentioned earlier, treatment discontinuation was not required; the same was described in literature as well [25,26]. However, we underline the need of close monitoring of treated patients, critical for early diagnosis of potential serious adverse events, such as severe hypokalemia or opportunistic infections. Notably, natural ACTH (Acthar gel) is not available in Greece and we therefore used the synthetic analogue tetracosactide (Synacthen) instead.

Our study has limitations: sample size is relatively small even regarding a limited population as KTx recipients, study design is retrospective and, most importantly, there is a significant diversity among patients and treatment options they received. Thus, baseline characteristics and treatment efficacy are difficult to categorize and evaluate, in a scientific field where randomized control trials are difficult, even impossible to implement. However, our findings emphasize that KTx in patients with primary FSGS still enquires several issues requiring solutions. Successful transplantation is vital for their survival, considering that recurrence rate is high. Some patients respond to standard treatment with plasmapheresis and rituximab poorly and new, arising therapeutic options are imperative. Although abatacept was put aside after initial enthusiasm, the drug may have a place in treating patients with B7.1 positive biopsy staining. ACTH, once replaced by oral steroids, has resurfaced as perhaps the most promising option in refractory disease. New agents including ofatumumab, a second generation anti-CD20 antibody [24,52,53,54] and an anti-CD40 antibody [16] arise as novel therapies. Consequently, despite these challenges, transplantation should not be withheld in those patients; a cohort who is comprised mostly of children and young adults, hoping for progress regarding treatment and better outcomes in the near future.

## Figures and Tables

**Table 1:** Comparison of demographics and baseline characteristics between KTx recipients with and without focal segmental glomerulosclerosis (FSGS)recurrence in the graft.

	Recurrence	No Recurrence	*p* Value
N	26 (57)	20 (43)	
Gender (male)	16 (67)	13 (65)	0.809
Age at transplantation (years)	33.8 (±13)	41.4 (±14.3)	0.067
Age at onset (years)	21.8 (±13)	27.7 (±14.3)	0.157
Time from diagnosis to ESRD (months)	61.4 (15.4–110.7)	111.2 (51–168.4)	0.038
Time on RRT (months)	30.3 (12–69.8)	27.2 (13.1–74.5)	0.622
Donor age (years)	53 (±11.6)	54.8 (±13.6)	0.654
Donor (living/deceased)	12/14 (46/54)	13/7 (65/35)	0.203
HLA mismatches	2.5 (±1.2)	2.6 (±1.3)	0.941
Delayed graft function	10 (39)	3 (15)	0.079
Number of transplantations			
1st	23 (89)	20 (100)	0.110
2nd	2 (8)	0	0.204
3rd	1 (4)	0	0.375
Prophylactic plasmapheresis	16 (62)	18 (90)	0.029
eGFR at year 1(mL/min/1.73 m^2^)	45 (±18)	53 (±15)	0.104
Follow-up time (months)	33.5 (23–57.8)	33 (18.2–140.6)	0.580
Graft loss due to any cause	15 (58)	0 (0)	
Time to ESRD (months)	31.5 (15.4–73.1)	NA	

Values are presented as number (%), mean (±SD) or median (IQR). ESRD: End Stage Renal Disease, RRT: Renal Replacement Therapy, HLA: Human Leukocyte Antigens, eGFR: estimated glomerular filtration rate.

**Table 2:** Outcomes of patients with FSGS recurrence in the graft following immunosuppressive therapy.

	Complete Remission	Partial Remission	No Remission	*p* Value
N (%)	7 (27)	11 (42)	8 (31)	
Early/late recurrence	6/2 (86/29) *	11/3 (79/21) *	6/2 (80/20)	
Time to recurrence (months)				
Early	0.72 (±0.41)	0.56 (±0.36)	0.36 (±0.18)	0.24
Late	13.5 (±6.36)	16.6 (±8.3)	16 (±5.66)	0.677
Maximal proteinuria (g/24 h)	6.1 (3.71–9.15)	9.5 (5.9–16.52)	10.3 (5.8–12.37)	0.434
Prophylactic plasmapheresis	5 (71.4)	7 (63.6)	4 (50)	0.684
Therapeutic plasmapheresis	7 (100)	11(100)	8 (100)	
Number of plasmaphereses	16 (14–19)	49 (24–61)	14 (12–31)	0.546
Rituximab	2 (18.6)	5 (45.5)	1 (12.5)	0.304
Abatacept	0	0	1 (12.5)	0.31
ACTH	1 (14.3)	2 (18.1)	1 (12.5)	0.94
Time to remission (months)	3 (±1.79)	4.4 (±2.25)	NA	0.547
Graft loss due to FSGS	0	4 (36.3)	7 (87.5)	0.025
Time to ESRD due to FSGS (months)	NA	36.9 (29.74–44.82)	18.3 (13.1–25.3)	0.389
Graft loss due to any cause	2 (25)	6 (54.5)	7 (87.5)	0.122
Time to ESRD	88.2 (52.15–124.38)	33.1 (17.87–57.73)	18.3 (13.1–25.3)	0.745

Values are presented as number (%), mean (±SD) or median (IQR). ACTH: adrecocorticotrocotropic hormone, ESRD: End Stage Renal Disease, NA: not applicable. * One patient in the first and three in the second group suffered both an early and a late recurrence.

## Data Availability

Data are not available publicly due to ethical restrictions.
